# Clinical characteristics and surgical treatments of primary hepatic angiosarcoma

**DOI:** 10.1186/s12876-021-01743-3

**Published:** 2021-04-07

**Authors:** Lei Jiang, Lijun Xie, Ge Li, Hang Xie, Zhao Fang, Xinran Cai, Yanling Chen

**Affiliations:** 1 Department of Hepatobiliary Surgery and Fujian Institute of Hepatobiliary Surgery, Fujian Medical University Union Hospital, NO. 29, Xinquan Road, Fuzhou City, Fujian Province China; 2 Department of Intervention Therapy, Fujian Medical University Union Hospital, Fujian, 350001 China; 3 Department of Ultrasonic Image, The First Affiliated Hospital of Fujian Medical University, Fuzhou, 350005 Fujian China; 4 Key Laboratory of The Ministry of Education for Gastrointestinal Cancer, Fujian Medical University, Fuzhou, 350108 Fujian China

**Keywords:** Primary hepatic angiosarcoma, Clinical characteristics, Surgical treatments, Hepatic lobectomy, Transarterial chemoembolization

## Abstract

**Purpose:**

Primary hepatic angiosarcoma is a very rare and highly malignant tumor with poor prognosis. It is difficult to diagnose because of the lack of typical clinical features, and the treatment protocols for PHA are also not clear. Therefore, this study wants to find out the clinical characteristics and surgical treatments of primary hepatic angiosarcoma.

**Methods:**

Among 8990 patients diagnosed with primary malignant tumor of the liver from January 2000 to December 2019 in our hospital, only four patients were diagnosed with primary hepatic angiosarcoma. The demographics, clinical manifestation, past history, serology test results, MRI features, pathology, treatment modality and prognosis of four patients were collected and analyzed.

**Results:**

Three of four patients had no clinical symptoms, while one patient's symptom was abdominal pain. The levels of tumor markers of all four patients were within the normal reference range and serological tests were negative for hepatitis B and C virus. The MRI imaging findings of all four patients were mixed mass with highly disordered vascular characteristics. All four patients were misdiagnosed preoperatively. One patient who underwent hepatic lobectomy was still alive for about 18 months after surgery. One patient who underwent hepatic lobectomy has survived for only 6 months due to severe pneumonia. The other two patients who received transarterial chemoembolization survived 16 months and 11 months respectively.

**Conclusion:**

The clinical symptoms of primary hepatic angiosarcoma are not typical, and primary hepatic angiosarcoma is easily misdiagnosed. The typical imaging manifestations are structural disorder and heterogeneous tumor. Hepatic lobectomy and transarterial chemoembolization may be important surgical treatments to improve the prognosis of patients.

## Introduction

Primary hepatic angiosarcoma (PHA) is a very rare malignant mesenchymal tumor. It is caused by the invasive growth of vascular or lymphatic endothelial cells, which accounts for 2% of primary liver malignant tumors [1]. The prognosis of PHA is poor, and no matter what treatment is used, very few patients can survive for more than 2 years [2, 3]. It is difficult to diagnose the disease because of the lack of typical symptoms and the absence of specific markers. The diagnosis of many cases depends on pathological diagnosis.

The best treatment for PHA is not clear. Surgery is the only treatment that offers potential treatment and realizes the long-term survival of tumor patients [4]. In the past two decades, great advances in surgical techniques have resulted in a significant reduction in mortality in patients undergoing hepatectomy and liver transplantation [5]. Yet the study of prognosis after hepatectomy is limited by a small number of patients with long-term treatment [6]. Liver transplantation which does not improve the prognosis of patients is not recommended [7, 8]. On the other hand, transcatheter intervention therapy, including embolization and chemoembolization, has been widely used in patients with unresectable liver cancer [9]. Transarterial chemoembolization (TACE) can be explored as an alternative surgical treatment for unresectable primary and metastatic liver sarcomas. Transcatheter embolization (TAE) which can stop bleeding and control tumors is also considered to be an effective treatment when hepatic angiosarcoma ruptures [10].

Although PHA is a highly malignant and rapidly progressing endothelial tumor, we are still unfamiliar with its clinical characteristics and surgical treatments. Among 8990 patients diagnosed with primary malignant tumor of the liver from 2000 to 2019 in our hospital, only 4 patients with PHA were selected as the research objects. This paper reports the clinical characteristics, imaging diagnosis, surgical treatments and prognosis of the 4 cases of PHA.

## Patients and methods

### Patients

We conducted retrospective case reviews of patients diagnosed with PHA in our hospital. There were four cases (0.044%) of PHA out of 8990 primary liver malignant tumor patients from January 2000 to December 2019 in our hospital. All four cases were confirmed as PHA by histopathology. The patients were followed up until September 2020. This study was approved by the institutional ethics committee of Fujian Medical University Union Hospital, and the informed consent was signed by all participants.

### Clinical evaluation of tumor

The following information was obtained from the patient's medical records: age, gender, environmental history, clinical symptoms, laboratory profiles including whole blood cell count, liver function tests, carcinoembryonic antigen (CEA), carbohydrate antigen 19–9 (CA199) and alpha fetoprotein (AFP), hepatitis B and C profile, MRI imaging features, pathology and treatment methods. The liver function was classified by Child–Pugh grade, and the grading criteria referred to the literature report of Pugh RN [11]. Indocyanine green retention rate at 15 min (ICGR15) was tested for liver function preparation ability [12]. The calculation method of residual functional residual liver volume (RFLV) refers to the research literature of Urata K [13].

### Follow-up

The patients were followed up every 3 months after the operation. Overall survival (OS) was defined as the period of time from the initial diagnosis of PHA to death from any cause or last follow-up.

## Results

### Clinical characteristics.

The baseline clinical characteristics of the patients are shown in Table 1. There were two males and two females (ratio 1:1). The median age was 55 years (range 24–75 years). Most patients had no obvious clinical symptoms, while one patient showed persistent pain in the right upper abdomen with reflex pain in the right back. One patient had a history of long-term exposure to chemicals as a truck driver in China Zijin Mining Ltd. Co. The number of liver masses in all cases was single. There were no extrahepatic distant metastases in all cases. One patient had the rupture and bleeding of angiosarcoma. All cases in this study were misdiagnosed as liver abscess, benign hepatic nodules, primary liver cancer and hepatic hemangioendothelioma respectively.

**Table 1:** The clinical characteristics of patients with primary hepatic angiosarcoma

Patient	Sex/age	Clinical symptom	Chemical exposure	No. of hepatic mass	Metastases	Tumor rupture	Misdiagnose	Treatment	OS (months)	Cause of death
1	M/24	RUA pain	Yes	Single	No	No	Yes	Segment hepatectomy	A 18	No
2	M/75	None	No	Single	No	No	Yes	Segment hepatectomy	E 6	Severe pneumonia
3	F/59	None	No	Single	No	No	Yes	Four TACE interventions	E 16	PD
4	F/63	None	No	Single	No	Yes	Yes	Two TACE interventions	E 11	PD

M, male; F, female; RUA, right upper abdomen; TACE, transarterial chemoembolization; OS, overall survival; A, alive; E,expired; PD, disease progression

Table 2 shows the laboratory profile of patients with PHA in this study. There was anemia in one patient whose hemoglobin value was 85 g/L. Aspartate aminotransferase (AST) and alanine aminotransferase (ALT) in one patient were higher than 40 IU/L. All patients were in Child–Pugh class A by liver function test. Serologic tests for hepatitis B and C were carried out in four patients and all were negative. AFP, CEA and CA19-9 of all patients were within the normal reference range.

**Table 2:** The laboratory profile of patients with primary hepatic angiosarcoma

Patient	WBC (10^9^/L)	Hb (g/L)	Plt (10^9^/L)	TBIL (umol/L)	AST/ALT (IU/L)	PT (INR/s)	AFP/CEA/CA19-9 (ng/ml)/(ng/ml)/(U/ml)	Hepatitis B and C	RFLV (%)	ICGR-15(%)
1	8.03	145	128	8.1	58/101	0.94/12.4	5.52/1.4/7.88	Negative	70.69	2.34
2	9.44	114	310	10.3	46/36	0.97/12.8	3.77/4.3/22.84	Negative	58.13	5.32
3	8.6	85	233	12.4	24/31	1.18/15.0	1.33/0.7/16.92	Negative	23.92	38.63
4	6.72	127	165	15.7	34/37	1.06/13.8	1.63/1.4/12.28	Negative	31.87	31.45

WBC, white blood cell; Hb, hemoglobin; Plt, platelet; TBIL, Total bilirubin; AST, aspartate transaminase; ALT, alanine transaminase; PT, Prothrombin time; AFP, a-fetoprotein; CEA, carcinoembryonic antigen; RFLV, residual functional residual liver volume; ICGR15, indocyanine green retention rate at 15 min

### The imaging findings

**Fig. 1:**
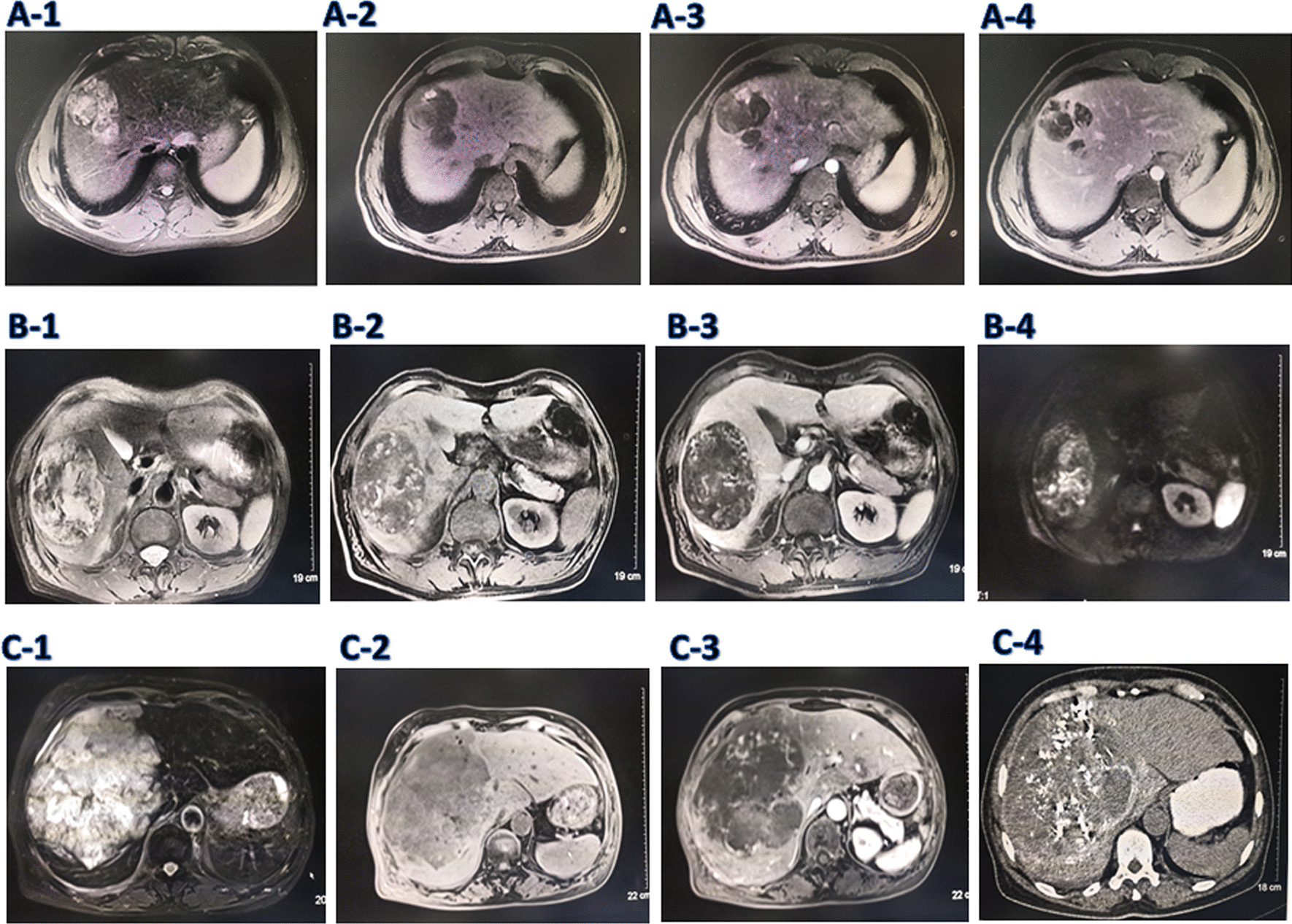
The imaging findings of PHA patients 1, 2 and 3. Patient 1. An irregular space occupying lesion with a size of 5.7 × 4.3 × 4.3 cm could be seen in the right liver. ***A-1***. On T2WI, there was a high signal, septum and mixed signal shadow; ***A-2***. On T1WI, most of them were hypointense, and a high signal nodular shadow was seen on the edge; ***A-3***. On the contrast-enhanced scan, there was a little nodular enhancement; ***A-4***. In portal phase, the enhancement of marginal nodule disappeared, and the septal stroma gradually strengthened. Patient 2. A tumor about 6.7 × 8.8 × 8.0 cm in size was found in the right liver. ***B-1***. On T2WI, there was mixed high and low signal intensity; ***B-2***. On T1WI, most of the tumors were hypointense and a few were patchy hyperintense; ***B-3.*** Irregular strip and progressive enhancement were found in the tumor. ***B-4***. MR diffusion weighted imaging showed that most of the tumors showed mixed restricted diffusion. Patient 3. A tumor about 15 × 14 × 13 cm in size in the whole right liver, part of which was located in the left liver. ***C-1***. On T2WI, most of the tumors showed high signal intensity, a few of them were extremely high signal, and a small amount of low signal. ***C-2***. On T1WI, the tumor showed hypointensity. ***C-3.*** On a contrast-enhanced scan, the tumor showed irregular strip reinforcement. ***C-4***. One month after TACE, a CT scan of the liver showed that there was a lot of lipiodol accumulation and deposition in hepatic angiosarcoma

**Fig. 2:**
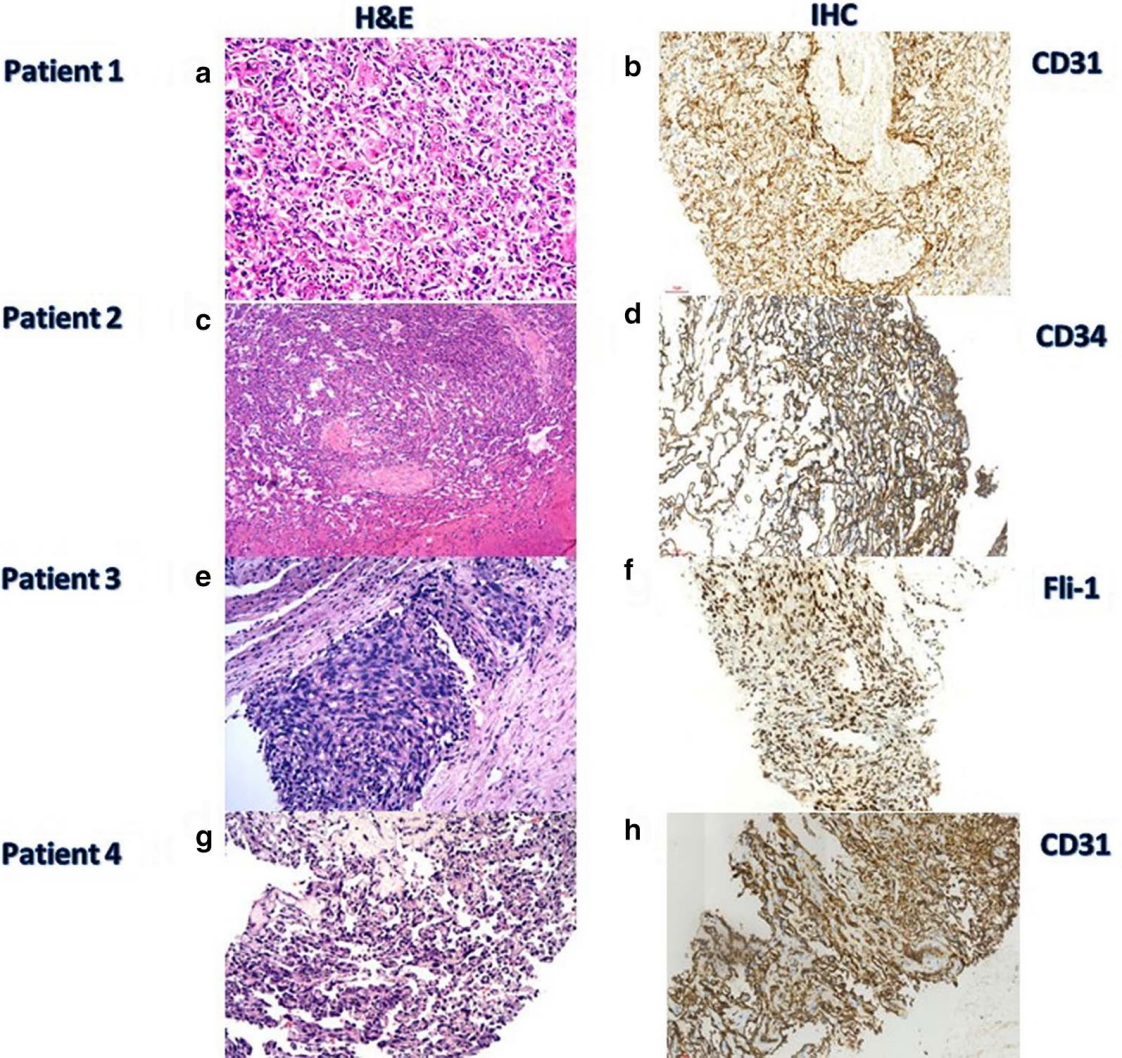
Pathologic findings in patients 1, 2, 3 and 4. Pathology findings in patient 1 [**a**, hematoxylin and eosin (H&E) stain, × 400; **b**, immunohistochemical stain of CD31, × 200] showed the endothelial cells with mild to moderate heterotype, hyperchromatic nuclei, and CD31 positive. Pathology findings in patient 2 (**c**, H&E stain, × 100; **d**, immunohistochemical stain of CD31, × 200) show that mitotic were found to anastomose with each other to form vascular like structures, favoring angiosarcoma and CD34 positive. Pathology findings in patient 3 (**e**, H&E stain, × 200; **f**, immunohistochemical stain of FLi-1, × 200) show malignant spindle cell tumor, favoring angiosarcoma and FLi-1 positive. Pathology findings in patient 4 (**g**, H&E stain, × 200; **h**, immunohistochemical stain of CD31, × 200) show malignant spindle cell tumor, favoring angiosarcoma and CD31 positive

**Fig. 3:**
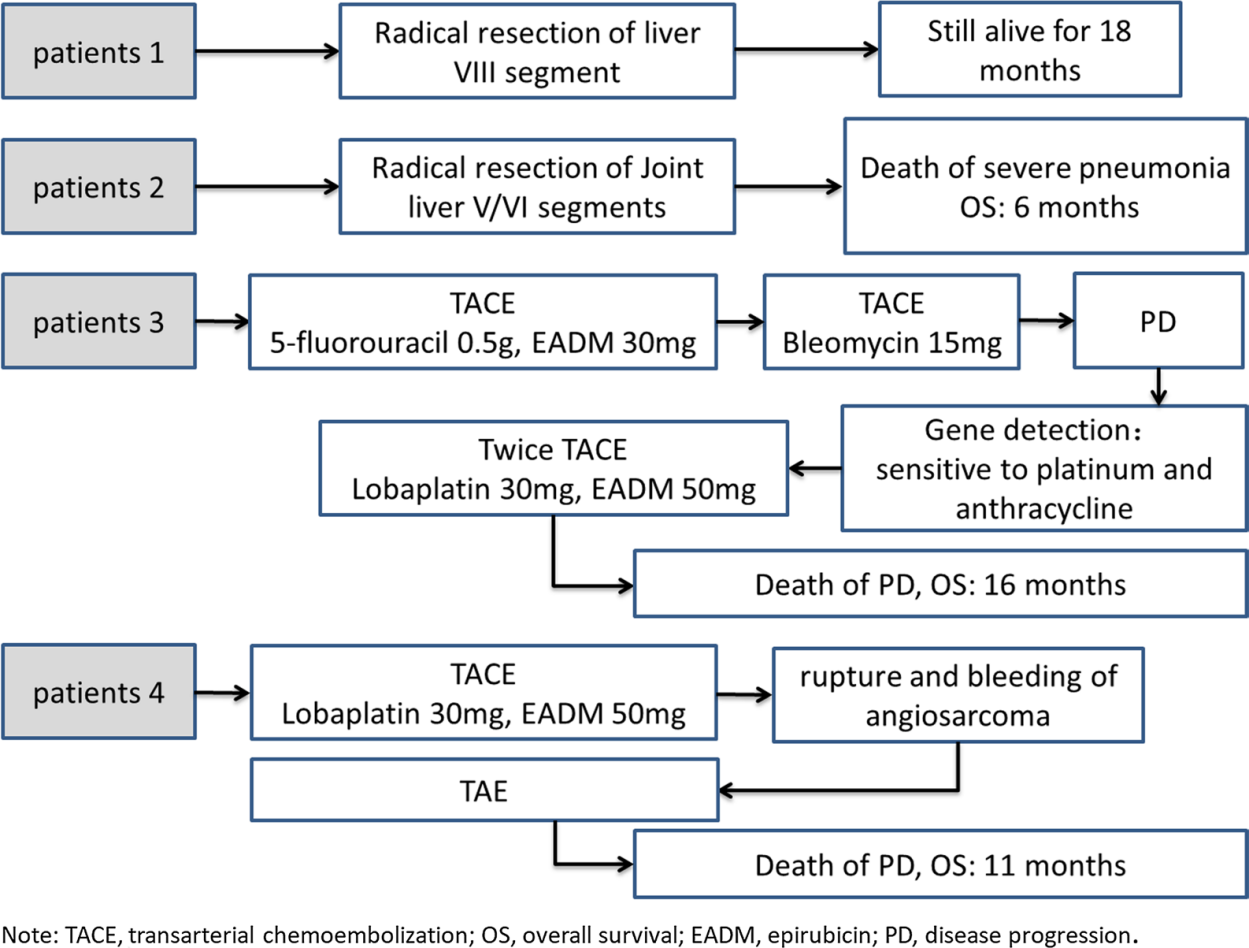
Surgical treatment outcomes for the individual patients 1, 2, 3 and 4

**Fig. 4:**
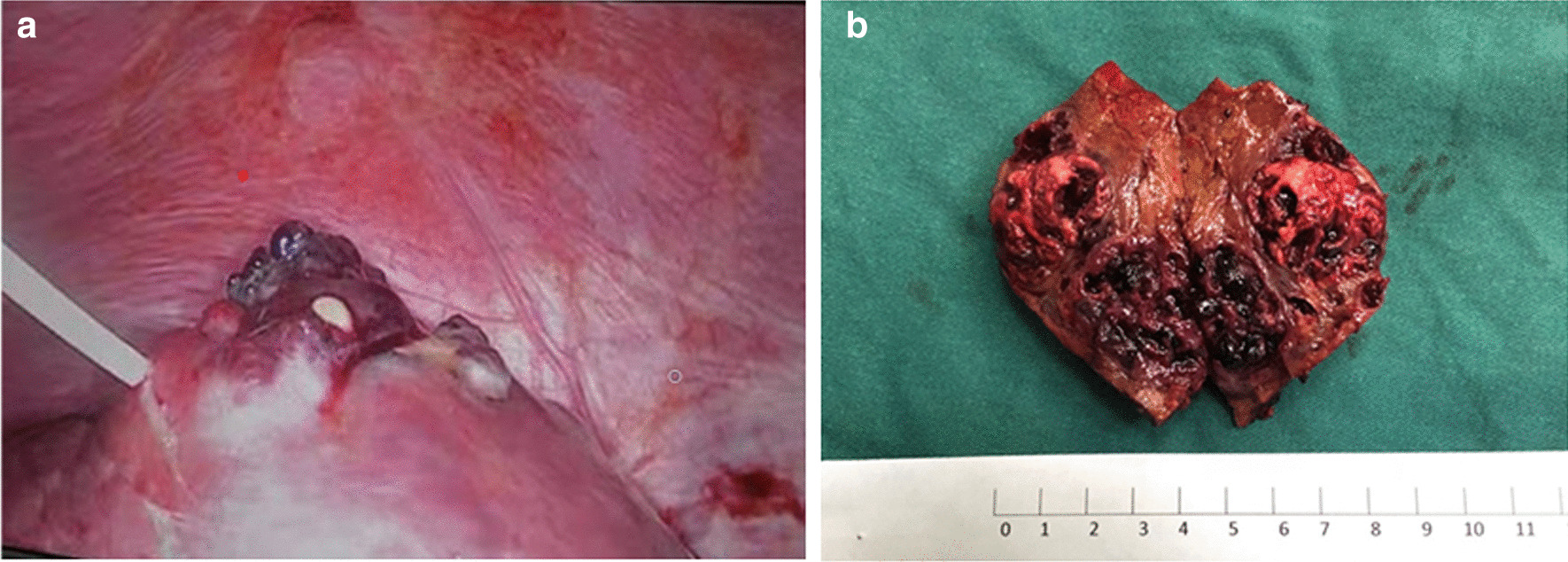
Intraoperative findings and postoperative specimen of patient 1. **a** During the operation in the local hospital, a hemangioma like mass was found in the upper part of the right anterior lobe of the liver, protruding on the surface of the liver, and the liver tissue around the tumor was enlarged. The purulent fluid mixed with blood outflow could be seen in the puncture biopsy. **b** After the operation in our hospital, the gross tumor specimen was dissected and found that the shape of the tumor was irregular, interlaced with hemorrhage and necrosis, and there was a large cavity filled with clots in the tumor

The enhanced MRI of liver is very important for the differential diagnosis of intrahepatic tumors. The MRI features of the patients with PHA are shown in Fig. 1 All the tumors were located in the right liver. Two patients (patients 3 and 4) had huge tumors, and the tumor sizes were 14 cm and 12.3 cm respectively, occupying the whole right liver, and a small part of one case extended to the left liver. In the other two patients (patients 1 and 2), the tumor sizes were 5 cm and 7.8 cm respectively, located at 8th segment and the junction of the 6th/5th segments of the liver. On T2-weightes image, all tumors showed mixed hyperintensity, and some cases showed internal septation (see Fig. 1A-1, B-1, C-1). On unenhanced T1-weightes image, all patients showed low signal intensity (see Fig. 1A-2, B-2, C-2). In one patient, there was a small nodular high signal on the edge of low signal (see Fig. 1A-2), and another patient had patchy high signal mixed with low signal (see Fig. 1B-2). There may be intralesional haemorrhage. MR diffusion weighted imaging showed that most of the tumors showed mixed restricted diffusion (see Fig. 1B-4). On contrast-enhanced scan, most of the patients showed irregular enhancement within the tumor (see Fig. 1A-3, B-3, C-3), and one patient showed a nodular enhancement on the edge (see Fig. 1A-3). On portal phase, most of the patients showed disorderly, heterogeneous enhancement, but not completely filled (see Fig. 1A-4). And one patient showed nodule enhancement disappeared, the septum strengthened gradually.

### Pathology

We evaluated all tumors under microscope, analyzed the pathological characteristics, and confirmed the pathological diagnosis. Hematoxylin and eosin stain of all patients showed that the endothelial cells with mild to moderate heterotype, hyperchromatic nuclei and mitotic were found to anastomose with each other to form vascular like structures, suggesting angiosarcoma (See Fig. 2). Hemorrhage and necrosis were seen in Patient 1. Positive immunohistochemical staining for CD31 and CD34, favoring angiosarcoma, was observed in all patients. In addition, the immunohistochemical staining for FLI-1 was positive in patients 2 and 3, the immunohistochemical staining for FVIII was positive in patient 1, and the immunohistochemical staining for Ki67 was positive in patients 3 and 4 (See Table 3). All these pathological results indicate that the four patients are angiosarcoma.

**Table 3:** Immunohistochemical staining results in patients 1, 2, 3 and 4

Patient no	Immunohistochemical stain	
	Positive	Negative
1	CD31, CD34, FVIII	GPC-3, Heppar-1, CK19, CK7, CK
2	CD31, CD34, FLi-1, Factor8	AFP, GPC-3, Heppar-1, CK19, CK7, CEA, CD10
3	CD31, CD34, FLi-1, Ki67	S-100, SMA, Desmin, HMB45, CK
4	CD31, CD34, Ki67	D2-40, WT-1, CK

### Surgical treatment outcomes

It is generally believed that patients with ICGR15 < 10% of Child-A can tolerate extensive hepatectomy with four liver segments; when ICGR15 is 30–39% in Child-A patients, only a limited small amount of hepatectomy can be performed [14]. The ICGR-15 results of two patients (patients 1 and 2) in this study were 2.34% and 5.32% respectively (See Table 2), so they performed radical resection of liver VIII segment and Joint liver V/VI segments respectively. The ICGR-15 results of the other two patients (patients 3 and 4) were 38.63% and 31.45% respectively (See Table 2), so they performed TACE for treatment.

Figure 3 shows surgical treatment outcomes for the individual patients.

Patient 1, who was diagnosed with liver abscess, performed the operation of laparoscopic drainage at a local hospital. It could be seen that a hemangioma like mass was seen in the upper part of the right anterior lobe of the liver, protruding on the surface of the liver (see Fig. 4a). Subsequently, the patient who was referred to our hospital underwent radical resection of liver VIII segment. Postoperative tumor specimen showed an irregular tumor, which was 5.5 × 4.3 cm in size, staggered with hemorrhage and necrosis, and a large cavity filled with clots could be seen in the tumor (see Fig. 4b). The patient was still alive, and survival was observed for 18 months after diagnosis.

Patient 2 underwent radical resection of Joint liver V/VI segments in our hospital. Unfortunately, the patient was diagnosed with severe pneumonia after the operation. The patient was sequentially transferred to the ICU of our hospital and Fuzhou Pulmonary Hospital for further treatment. Finally, the patient died of severe pneumonia and he survived for 6 months after diagnosis.

Patient 3 performed TACE under local anesthesia, in which the tumor was injected with 5-fluorouracil 0.5 g and EADM 30 mg. A month later, a liver CT scan showed that there was a lot of lipiodol accumulation and deposition in angiosarcoma (see Fig. 1C-4). The patient performed TACE again, in which the tumor was injected with Bleomycin 15 mg. Due to disease progression, the tumor tissue was sent for genetic detection which showed that the tumor was sensitive to platinum and anthracycline chemotherapy drugs. Then the patient performed twice TACEs, in which the tumor was injected with lobaplatin 30 mg and EADM 30 mg. Finally, the patient died of disease progression and he survived for 16 months after diagnosis.

Patient 4 performed TACE under local anesthesia, in which the tumor was injected with lobaplatin 30 mg and EADM 30 mg. Unfortunately, rupture and bleeding of angiosarcoma about half a year later, so TAE was performed under emergency local anesthesia. Finally, the patient died of disease progression and he survived for 11 months after diagnosis.

## Discussion

PHA is a rare and highly malignant tumor derived from mesenchymal tissue. It comes from vascular endothelial cells of the liver, also known as vascular endothelial sarcoma, Kupffer cell sarcoma and malignant hemangioendothelioma [15, 16]. Because of its low incidence rate, and lack of specific clinical manifestations, laboratory and imaging examinations, it is often difficult to diagnose in clinic. It is easy to be misdiagnosed as hepatic hemangioma, primary hepatocellular carcinoma and others [17]. In patient 1 of this study, the patient was misdiagnosed as liver abscess because of concomitant fever. Subsequently, the patient underwent laparoscopic drainage of liver abscess. During the operation, hemangioma like mass and pus like substances were found in the puncture tumor. So the patient was misdiagnosed as hepatic hemangioma with infection. In patient 2, the patient was misdiagnosed as “benign hepatic nodules, such as inflammatory pseudotumor and atypical hemangioma" in the local hospital. During the follow-up, a small amount of rupture and bleeding occurred in the liver tumor. So the diagnosis was considered as liver malignant tumor. In patient 3, the patient was misdiagnosed as primary liver cancer preoperatively. In patient 4, the patient was misdiagnosed as a benign liver tumor, such as hepatic hemangioendothelioma. Therefore, it is necessary to standardize the diagnostic criteria for hepatic angiosarcoma, including medical history, symptoms, signs and imaging examination to improve the diagnostic accuracy of the disease.

The etiology of most PHA is unknown. Some scholars found that it may be related to long-term exposure to bismuth dioxide colloid, vinyl chloride (VC), arsenic and other factors [18–20]. The most significant association was with VC [21]. In a comprehensive review, kielhorn et al. [22] Found that exposure to VC was associated with 197 cases of hepatic angiosarcoma published before the 1990s, and with the technical reform of VC industry, the reports of hepatic angiosarcoma in new workers exposed to VC are much less. It has also been reported that PHA is associated with K-ras and p53 mutations [23]. In patient 1 of this study, the patient had a history of long-term exposure to chemicals as a truck driver in China Zijin Mining Ltd. Co. The other three patients (patients 2,3 and 4) had not been exposed to carcinogens. Most patients often have nonspecific clinical symptoms, such as abdominal pain, weakness, fatigue, weight loss, hepatomegaly, ascites and jaundice [1]. Tumor markers, including AFP, CA19-9 and CEA, are usually not elevated. In patient 1, the first symptom of the patient was right upper abdominal pain with a low fever. The other three patients (patients 2,3 and 4) were found in the imaging examination without obvious clinical symptoms. In all the above patients, tumor markers such as AFP, CEA and CA199 were in the normal range.

According to tumor morphology, PHA can be divided into four types: diffuse micro nodule type, diffuse multiple nodule type, massive type and mixed type [24]. In the four patients we studied, all of them were single mass type, in which patient 3 and patient 4 were single giant mass type. But some scholars believe that the most common type of PHA is diffuse multiple nodules [25]. Imaging examination is a necessary auxiliary means for the diagnosis of PHA, which can make the initial diagnosis of localized or suspected non liver cancer, and provide reference for subsequent diagnosis and treatment. In contrast-enhanced MRI or CT, PHA is a progressive enhancement of vascular tumor, so it may be misdiagnosed as hepatic hemangioma [26, 27]. But if you identify the image carefully, you can still differentiate it from hepatic hemangioma. Due to the highly disordered vascular characteristics and heterogeneity of PHA, the vascular lesions in the tumor are intertwined with each other [28], resulting in the tumor presenting as a mixed mass on MRI. 80% of hepatic angiosarcoma showed heterogeneous branching and stent like structures, which is a typical feature different from hepatic hemangioma [29]. In the 4 patients in this study, MRI images showed heterogeneous high and low mixed shadows, structural disorder, and some of them were strip like. An enhanced scan showed the "centrifugal" enhancement of the tumor from the inside to the outside. In portal venous phase, the tumors were not evenly filled. This is obviously different from the benign hemangioma of the liver. This is consistent with Perry J. Pickhardt 's study [30].

Because of the low incidence of PHA, there are no standard treatment guidelines for PHA. Complete surgical resection is the preferred treatment, which may also lead to long-term survival for some patients [31, 32]. The prognosis of PHA is very poor. Without treatment, most patients die within six months of diagnosis [15], and only 3% of patients live longer than 2 years [20]. Therefore, for PHA patients who can undergo hepatectomy after pre-operative evaluation, active surgical resection can significantly improve the survival time of patients. Zheng YW 's study report success with complete excision, extending the median survival to 17 months [10]. In patient 1 of our studies, a radical resection of liver VIII segment was performed in our hospital. The patient is still alive for 18 months after diagnosis. In patient 2, the patient underwent complete excision in our hospital. Unfortunately, the patient developed severe interstitial pneumonia after surgery, and so the patient survived only 6 months. However, there are also some patients who can not be resected after tumor evaluation once they are found. In some cases, the initial manifestation is life-threatening bleeding caused by spontaneous tumor rupture [15]. Studies have confirmed that [33] TAE is safe and can play a role in the selective treatment of unresectable primary and metastatic hepatic sarcoma. Emergency embolization of PHA rupture is effective in preventing bleeding, stabilizing the patient's condition and controlling the blood supply of tumor [10]. At present, the transcatheter liver guided therapy for hepatic sarcoma has not been considered as a standard treatment method, and there is no consistent guideline [34]. In patient 3, the tumor was huge, occupying the entire right liver and partially located in the left liver. Preoperative assessment showed that the remaining liver volume was not enough to allow surgical treatment, so we performed four TACE operations at different time points, combined with local platinum anthracycline chemotherapy. The patient eventually survived 16 months. In patient 4, because of a huge tumor, we performed TACE on the patients. But the patient had tumor rupture and bleeding half a year after the operation, and then she underwent emergency TAE treatment. The patient eventually survived 11 months.

In conclusions, the clinical symptoms of PHA are not typical, and it is easily misdiagnosed. The etiology of most PHA remains unclear and may be related to exposure to certain toxic chemicals. The typical imaging manifestations are structural disorder and heterogeneous tumor. Hepatic lobectomy and TACE may be important surgical treatments to improve the prognosis of patients.

## Data Availability

All data generated or analysed during this study are included in this published article.

## Abbreviations

PHA: Primary hepatic angiosarcoma
TACE: Transarterial chemoembolization
TAE: Transcatheter embolization
CEA: Carcinoembryonic antigen
CA199: Carbohydrate antigen 19–9
AFP: Alpha fetoprotein
ICGR15: Indocyanine green retention rate at 15 min
RFLV: Residual liver volume
OS: Overall survival
AST: Aspartate aminotransferase
ALT: Alanine aminotransferase
